# Diet as an Exposure Source and Mediator of Per- and Polyfluoroalkyl Substance (PFAS) Toxicity

**DOI:** 10.3389/ftox.2020.601149

**Published:** 2020-12-04

**Authors:** Katherine Roth, Zunaira Imran, Wanqing Liu, Michael C. Petriello

**Affiliations:** ^1^Institute of Environmental Health Sciences, Wayne State University, Detroit, MI, United States; ^2^Department of Chemistry, Wayne State University, Detroit, MI, United States; ^3^Department of Pharmaceutical Sciences, College of Pharmacy, Wayne State University, Detroit, MI, United States; ^4^Department of Pharmacology, School of Medicine, Wayne State University, Detroit, MI, United States

**Keywords:** NAFLD, PPAR, diet, PFAS, hyperlipidemia

## Abstract

Per- and polyfluoroalkyl substances (PFAS) are ubiquitously found in the environment due to their widespread commercial use and high chemical stability. Humans are exposed primarily through ingestion of contaminated water and food and epidemiological studies over the last several decades have shown that PFAS levels are associated with adverse chronic health effects, including cardiometabolic disorders such as hyperlipidemia and non-alcoholic fatty liver disease. Perhaps the most well-established effects, as demonstrated in animal studies and human epidemiological studies, are the metabolic alterations PFAS exposure can lead to, especially on lipid homeostasis and signaling. This altered lipid metabolism has often been linked to conditions such as dyslipidemia, leading to fatty liver disease and steatosis. Western diets enriched in high fat and high cholesterol containing foods may be an important human exposure route of PFAS and may also act as an important modulator of associated toxicities. In fact, the chemical structure of PFAS resemble fatty acids and may activate some of the same signaling cascades critical for endogenous metabolism. In this review we aim to outline known dietary exposure sources of PFAS, describe the detrimental metabolic health effects associated with PFAS exposure, and focus on studies examining emerging interaction of dietary effects with PFAS exposure that further alter the dysregulated metabolic state.

## Introduction

Recently, per- and polyfluoroalkyl substances (PFAS) have become a popular component added to numerous industrial and consumer products used in everyday life (Center for Disease Control and Prevention, 2017). The family of PFAS chemicals are carbon-chain molecules that have been fully fluorinated. They are created by replacing all hydrogen bonds with fluorine bonds of at least one carbon atom (poly-) or of all carbon atoms (per-) in a substance, such that they form the perfluoroalkyl moiety C_n_F_2n+1_- (Buck et al., 2011). The carbon-fluorine bond is particularly strong and stable, making PFAS highly resistant to degradation. The combination of the chemical and thermal stability of PFAS, along with their lipophobic/hydrophobic properties, has led to the use of PFAS for their excellent surfactant properties in industrial and consumer products such as cookware, clothing, and carpets, as well as in aqueous film-forming foams (AFFs) used in fire-fighting (Clara et al., 2008; Trier et al., 2011; Nickerson et al., 2020). Long-chain PFAS, known as “legacy” PFAS, have been used in products for decades, with the two most studied legacy PFAS being perfluorooctane sulfonic acid (PFOS) and perfluoro octanoic acid (PFOA). Studies screening a wide range of consumer products have determined that PFAS are present at significant levels in the majority of products tested (Kotthoff et al., 2015).

The robust resistance of PFAS to degradation has also led to their bioaccumulation in the environment and in animals and humans (Wang et al., 2015). Legacy PFAS have been increasingly measured in environments across the globe, including water sources and wildlife food webs (Giesy and Kannan, 2001; Domingo and Nadal, 2019). In accordance with the bioaccumulation of PFAS in the environment, the U.S. Centers for Disease Control and Prevention (CDC) reported via data collected from the National Health and Nutrition Examination Survey (NHANES) 2003-2004 that legacy PFAS compounds are detectable in the blood of 98% of adult Americans (Center for Disease Control and Prevention, 2005; Calafat et al., 2007). The biological half-lives are long for many of the legacy PFAS. The elimination half-life from human serum has been calculated around 4 years for PFOA, 5 years for PFOS, and as great as 8.5 years for perfluorohexane sulfonate (PFHxS) (Olsen and Zobel, 2007). Due to the persistence of these chemicals, the growing amount of evidence that PFAS exposure can lead to adverse health effects in humans is alarming. Toxicity studies over the past decade have shown an association of PFAS exposure with immunotoxicity and chronic diseases including hepatic steatosis, cardiometabolic diseases, and cancer (DeWitt et al., 2012; Grandjean et al., 2012; Barry et al., 2013; Vieira et al., 2013; Liu H. S. et al., 2018). Because of the growing concern over the detrimental effects of legacy PFAS on the environment and on humans, legacy PFAS are being phased out and substituted by alternative, emerging PFAS. Emerging PFAS often feature shorter carbon chains or fluoroether replacements, such as perfluorobutane sulfonate (PFBS), ammonium perfluoro(2-methyl-3-oxahexanoate) GenX, and 4,8-dioxa-3H-perfluorononanoate (ADONA) (Lindstrom et al., 2011; Dodds et al., 2020). However, these emerging PFAS remain an area of active research and ongoing concern over their health impact on the environment and human population as well (Wang et al., 2015; Gomis et al., 2018).

## Exposure Routes of PFAS

**Figure 1:**
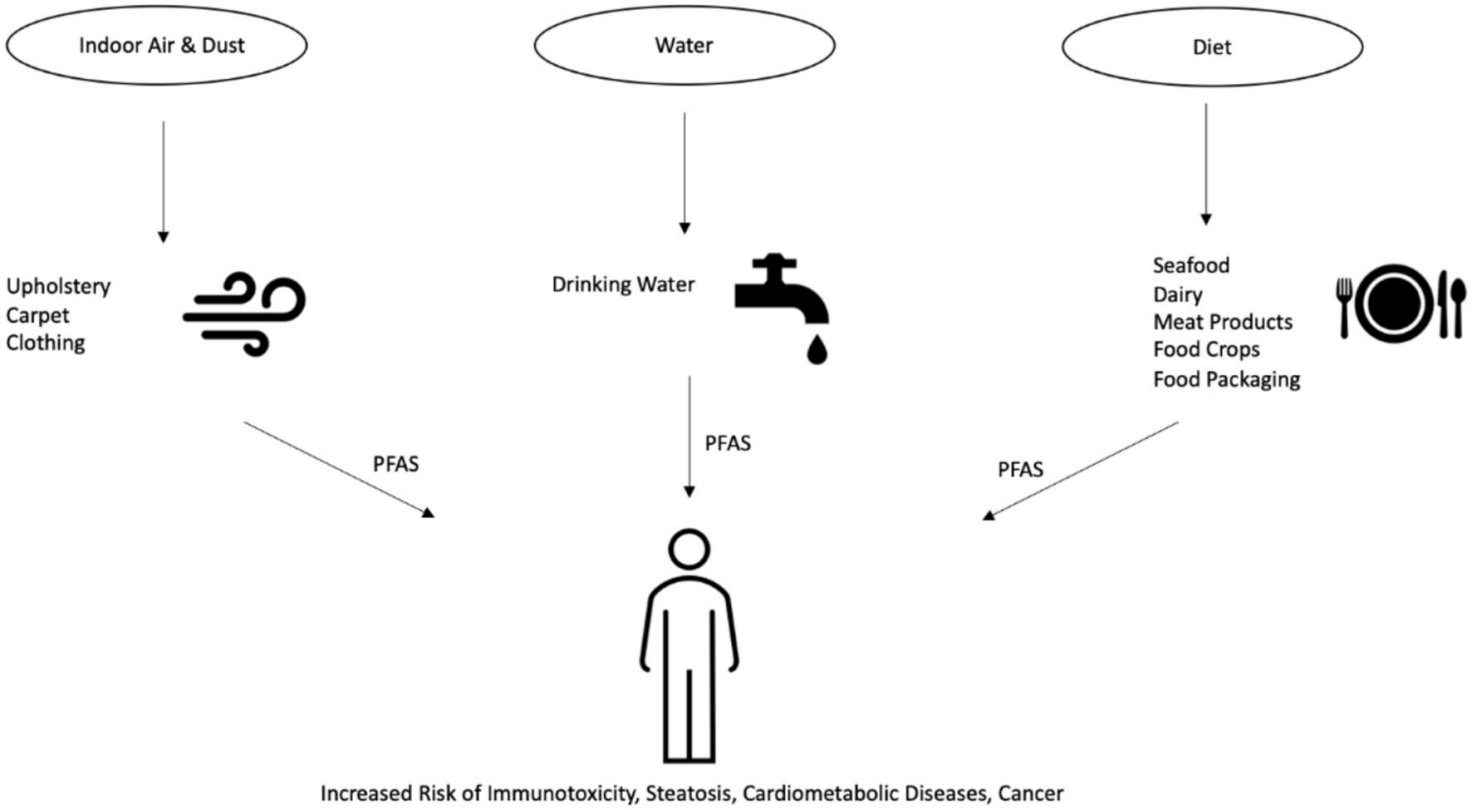
An overview of the various routes through which human exposure to PFAS can occur. First, PFAS can migrate from products such as carpet and clothing into the surrounding indoor air and dust. Second, PFAS enter the environment through production or waste streams and into the drinking water supplies. Lastly, PFAS present in animals, food crops, and food packaging can enter humans through their food and diets.

The U.S. Environmental Protection Agency (EPA) has published health advisories for PFOA and PFOS levels in drinking water set at 70 parts per trillion (ppt) (U.S. Environmental Protection Agency, 2016). In February 2020, the EPA announced its proposition to regulate PFOS and PFOA, which would establish official regulations on PFAS contaminant levels (U.S. Environmental Protection Agency, 2020). In Canada, the Canadian Federal-Provincial-Territorial Committee on Health and the Environment has set drinking water maximum acceptable concentrations of 600 and 200 ppt for PFOS and PFOA, respectively (Health-Canada, 2020). In other regions of the globe, the Heads of EPAs Australia and New Zealand (HEPA), as part of their PFAS National Environmental Management Plan (NEMP), have instated drinking water regulations at 70 ppt for PFOS and PFHxS and 560 ppt for PFOA (Heads of EPAs Australia and New Zealand, 2020). There are a variety of routes through which human exposure to PFAS are known to occur, including indoor dust and air, drinking water, and diet (Domingo and Nadal, 2017, 2019; Sunderland et al., 2019). These PFAS exposure routes are outlined in Figure 1. Although not as significant as drinking water and diet, studies have detected PFAS ingestion in indoor environments as a potential contributing factor to total body burden (Ericson Jogsten et al., 2012). PFAS are used in the manufacture of a wide variety of consumer products commonly found in households and offices, including carpets, upholstery, food packaging, and clothing (Guo et al., 2009; Zabaleta et al., 2016; Favreau et al., 2017; Robel et al., 2017). PFAS can migrate from said products and permeate into the indoor environment, collecting in air and dust that can be ingested by the environment's inhabitants. Inhalation of PFAS from these environments account for a detectable level of total human PFAS intake that can lead to detrimental health effects (Trudel et al., 2008; Harrad et al., 2010; Haug et al., 2011; Winkens et al., 2018). However, the average indoor environmental exposure is estimated to comprise only around 1% of total PFAS intake, whereas the main route of exposure to PFAS is through dietary intake (Ericson Jogsten et al., 2012). Numerous studies have shown that the consumption of drinking water is a robust exposure route for PFAS. PFAS have been detected in the surface water and drinking water in countries across the globe (Skutlarek et al., 2006; Takagi et al., 2008; Appleman et al., 2014; Schwanz et al., 2016). The exposure to PFAS is especially high in areas surrounding industrial and chemical manufacturing facilities, where multiple PFAS have been discovered contaminating nearby river and drinking water resources (Bach et al., 2017; Herrick et al., 2017). Populations exposed to PFAS-contaminated water sources have been associated with increased measured serum concentrations of PFAS (Emmett et al., 2006; Steenland et al., 2009; Frisbee et al., 2010; Seals et al., 2011). Further studies have established associations between PFAS exposure and adverse health effects of those exposed populations (Lopez-Espinosa et al., 2012; Barry et al., 2013; Darrow et al., 2013; Steenland et al., 2013). Although the focus of this review is on the association of PFAS exposure with hyperlipidemia and steatosis, there are other adverse outcomes that have been linked to PFAS. For example, in a 2008-2011 study of Mid-Ohio Valley adults exposed to PFAS through drinking water contamination, PFOA exposure was found to be associated with kidney and testicular cancer, as well as ulcerative colitis (Barry et al., 2013; Steenland et al., 2013). Another study of pregnant women from 2005 to 2010 exposed to PFAS through drinking water contamination in the same region found PFOA and PFOS levels positively associated with pregnancy-induced hypertension (Darrow et al., 2013). Moreover, in 2005-2006, as part of the C8 Health Project, it was found that in children exposed to PFAS through contaminated drinking water, PFOS, PFOA, and PFNA were associated with thyroid function impairment (Lopez-Espinosa et al., 2012). Of note, the use of granular activated carbon in water filtration systems has shown to be effective in lowering serum PFAS concentrations of the exposed populations (Bartell et al., 2010). However, continual exposure to even relatively low amounts of PFAS can still result in elevated body burdens and health risks (Post et al., 2012).

In addition to indoor environments and water, the greatest source of PFAS exposure is estimated to be through diet via the intake of contaminated foods (Ericson et al., 2008; Domingo and Nadal, 2017). Seafood consumption has been shown to be a key source of dietary PFAS. In 2018, the European Food and Safety Authority (EFSA) estimated that up to 86% of dietary exposure to PFAS from food comes from “fish and other seafood” (EFSA, 2018). Furthermore, PFAS have been measured in U.S. rivers and lakes, and data collected from the 2007–2014 U.S. NHANES found that higher serum PFAS levels are associated with greater fish and shellfish consumption (Stahl et al., 2014; Christensen et al., 2017). Although to a lesser extent than seafood, the EFSA also identified meat products and dairy products as contributors to dietary exposure to PFAS (EFSA, 2018). Studies across Europe and Asia have found contamination of dairy and meat products with PFAS are frequently detected but at low concentrations (Hlouskova et al., 2013; Barbarossa et al., 2014; Heo et al., 2014). In the U.S. FDA's Total Diet Study, which is an ongoing program monitoring levels of contaminants in the average U.S. diet, low levels of PFAS were detected in milk products, the highest being PFOA at 42 parts per trillion and perfluorononanoic acid (PFNA) at 39 parts per trillion (U.S. Food and Drug Administration, 2019). PFAS levels in cheeses were also measured at no < 200 ppt, with perfluorodecanoic acid (PFDA). In addition, PFAS have also been shown to enter and bioaccumulate in food crops after being taken up from the soil or water (Lechner and Knapp, 2011; Herzke et al., 2013; Blaine et al., 2014). Lastly, food contamination can occur indirectly through migration of PFAS from food packaging or cookware (Susmann et al., 2019). Studies have detected up to 46 PFAS in food packaging materials, such as microwave popcorn bags, at levels between 3.5 and 750 ng/g (Moreta and Tena, 2014; Zabaleta et al., 2017). However, no PFAS were detected in the popcorn after cooking, and whether or not this route of dietary exposure contributes significantly to the overall body burden remains inconclusive (Tittlemier et al., 2006; Vestergren et al., 2008; Jogsten et al., 2009; D'Hollander et al., 2010; Moreta and Tena, 2014).

## Impacts of PFAS on Metabolism and Metabolic Diseases

### Lipid Metabolism and Dyslipidemia

PFAS structurally resemble fatty acids and are known to activate peroxisome proliferator-activated receptors (PPARs), which elicit pathways that play key roles in lipid metabolism and adipogenesis (Vanden Heuvel et al., 2006; Wolf et al., 2012). Therefore, the ability of PFAS to disrupt lipid metabolism has been an active area of research. Studies over the past decades have revealed that dyslipidemia is the most evident metabolic outcome associated with exposure to PFAS. Epidemiological studies have shown that humans exposed to very high levels of PFAS, most frequently PFOS and PFOA, are positively associated with increased cholesterol and other serum lipids (Olsen and Zobel, 2007; Sakr et al., 2007; Costa et al., 2009; Steenland et al., 2009; Frisbee et al., 2010). Table 1 outlines many of these most recent studies on PFAS exposure in human epidemiological studies and the resulting endpoints related to metabolic disease. However, there has been variability in the classes of lipids seen to increase. For example, in a study of employees occupationally exposed to PFAS at the DuPont Washington Works manufacturing site in the Mid-Ohio Valley between 1979 and 2004, where employees had serum PFOA concentrations ranging from 0 to 22.66 ug/mL, an association was found between one ppm increase in serum PFOA and a 1.06 mg/dL increased total cholesterol (*P* = 0.011) (Sakr et al., 2007). However, no association was found with serum triglycerides. Additionally, in another study of DuPont workers, where measured PFOA serum levels ranged from 0.20 to 47.04 ug/mL, PFOA exposure was reported to significantly increase mean total cholesterol (*P* = 0.005) (Costa et al., 2009). On the contrary, in another study of employees exposed to PFAS at 3M plant locations in Antwerp, Belgium, in Decatur, Alabama, and in Cottage Grove, Minnesota, where serum PFOA levels ranged from 0.007 to 92.03 μg/mL (mean 2.21 μg/mL), PFOA was reported as positively associated with triglycerides (*P* < 0.0001), but not with total cholesterol (*P* > 0.05) (Olsen and Zobel, 2007). In residential communities exposed to environmental PFOA contamination from a chemical plant in West Virginia, Steenland et al. reported a positive correlation between PFOA and total cholesterol, with the odds of high cholesterol (>240 mg/dL) increasing 40–50% from the lowest quartile of PFOA and PFOS serum concentrations to the highest quartile (Steenland et al., 2009). When children and adolescents also exposed to PFAS drinking water contaminated from the DuPont chemical plant in West Virginia were evaluated as part of the C8 Health Project, it was reported that they also demonstrated elevated total cholesterol associated with both PFOA and PFOS (*p* < 0.0001), with a 4.6 and 8.5 mg/dL increase in total cholesterol between the lowest and highest quintiles of PFOA and PFOS, respectively (Frisbee et al., 2010). However, the significance of the positive association between PFAS and lipid levels for the general population has been disputed. The median serum concentration of PFOA in NHANES (2003-2004) participants was 4 ng/mL, which is around 1 to upwards of 3 orders of magnitude lower than those exposed occupationally or by contaminated environments (Calafat et al., 2007; Sakr et al., 2007; Costa et al., 2009; Nelson et al., 2010). Yet, when analyzing NHANES (2003-2004) participants, Nelson et al. found a positive association with total and LDL cholesterol despite the lower “background” PFAS exposure levels of the general U.S. population (Nelson et al., 2010). Another study of NHANES (1999-2008) participants observed PFAS exposure significantly associated with increased total and LDL cholesterol in adolescents in the U.S. at all exposure levels, including the lowest “background” exposure (Geiger et al., 2014). Although many studies support the correlation between PFAS exposure and lipid levels, the specific mechanisms of how PFAS alter lipid metabolism have not been completely elucidated. PFAS in serum are mainly proteinophilic as opposed to lipophilic, and studies have shown that albumin is the major carrier protein for PFAS with over 90% of PFAS bound to serum albumin in human blood (Han et al., 2003; Forsthuber et al., 2020). However, PFOA has not been shown to effect serum albumin levels, although it participates in the transportation of PFAS and fatty acids (Wang et al., 2013). Studies have shown that PFAS activate nuclear receptors like PPARs, and have been associated with changes in the expression of genes involved in lipid metabolism (Wolf et al., 2012; Fletcher et al., 2013). Furthermore, in a study by Liu et al. using samples from the 2-year Prevention of Obesity Using Novel Dietary Strategies (POUNDS) Lost study, plasma PFAS concentrations were primarily associated with blood lipids of specific subfractions that contain apoC-III, a known risk factor for coronary disease, suggesting that mechanistic research should focus on specific instead of total lipid levels (Mendivil et al., 2011; Liu G. et al., 2020).

**Table 1:** Summary of epidemiological studies linking PFAS exposure to metabolic diseases.

**Citation**	**Location**	**PFAS**	**Serum Concentrations**	**Physiological Endpoints**
Sakr et al. (2007)	U.S. (1979–2004, West Virginia)	PFOA occupational exposure	1.13 ug/ml (range, 0–22.66 ug/ml) PFOA	↑Total cholesterol has positive association with serum PFOA
Steenland et al. (2009)	U.S. C8 health project (2005–2006, Ohio and West Virginia)	PFOA and PFOS community exposure	80 ng/mL PFOA and 22 ng/mL PFOS	↑Total cholesterol and triglycerides have positive association with serum PFOA and PFOS
Gleason et al. (2015)	U.S. NHANES (2007–2010)	PFHxS, PFOS, PFOA, PFNA	11.0 μg/L PFOS, 1.2 μg/L PFNA, 3.5 μg/L PFOA, 1.8 μg/L PFHxS	↑ALT has positive association with all serum PFAS
Wang et al. (2014)	China	PFOA, PFOS, PFDA, PFNA, PFUnA, PFHxS	8.53 nM PFOA, 13.39 nM PFOS, 1.66 nM PFDA, 1.72 nM PFNA, 0.70 nM PFUnA, and 0.60 nM PFHxS	↑Metabolic syndrome risk associated with PFASs
Mora et al. (2018)	U.S. (2007–2010, Boston, children)	PFOS, PFOA, PFDeA	6.2 ng/mL PFOS, 4.3 ng/mL PFOA, and 0.3 ng/mL PFDeA	↑Plasma total cholesterol has positive association with PFOS, PFOA, and PFDeA
Jain and Ducatman (2019b)	U.S. NHANES (2011–2014)	PFHxS, PFOS, PFOA, PFNA, PFDA	Non-obeses: 2.2 ng/ml PFOA, 6.3 ng/ml PFOS, 0.21 PFDA, 1.41 PFHxS, 0.83 PFNA; Obese: 2.0 ng/ml PFOA, 5.5 PFOS, 0.18 PFDA, 1.24 PFHxS, 0.73 PFNA	↑Liver injury has positive association with serum PFOA, PFHxS, and PFNA (obese participants only)
Jin et al. (2020)	U.S. (2007–2015) children with diagnosed NAFLD	PFOA, PFOS, PFHxS	3.42 ng/ml PFOA, 3.59 ng/ml PFOS, and 1.53 ng/ml PFHxS	↑NAFLD severity has positive association with higher PFAS exposure

Although exposure of humans to PFAS is commonly associated with hyperlipidemia, the literature surrounding animal studies on PFAS exposure conversely report symptoms of hypolipidemia. Studies with rodents have generally reported that animals exposed to PFAS orally or through diet demonstrate serum cholesterol and triglyceride levels that are either decreased or not significantly altered (Haughom and Spydevold, 1992; Loveless et al., 2006; Martin et al., 2007; Qazi et al., 2010; Butenhoff et al., 2012). Table 2 outlines many of these important studies on PFAS exposure in animal models and the resulting endpoints related to metabolic disease. One study has reported, however, that while mice exposed to high levels of PFOA (30 mg/kg) displayed decreased serum triglyceride levels, lower levels of PFOA (0.3–10 mg/kg) demonstrated increased triglyceride levels (Loveless et al., 2006). In another study, Sprague-Dawley rats exposed to 20 mg/kg PFOA or 10 mg/kg PFOS daily for up to 5 days were reported to have decreased total serum cholesterol, downregulated cholesterol biosynthesis genes, and increased hepatocellular hypertrophy (Martin et al., 2007). In 2010, Qazi et al. exposed mice to dietary PFOA and PFOS for 10 days and similarly reported decreased total cholesterol, decreased serum triglycerides, and increased hepatocellular hypertrophy (Qazi et al., 2010). In wild-type mice receiving 1 or 5 mg/kd/day PFOA for 6 weeks, Nakagawa et al. reported decreased hepatic triglyceride levels and increased steatosis (Nakagawa et al., 2012). Further reports of hypolipidemic effects have been reported in studies of monkeys, where cynomolgus monkeys given 0.75 mg/kg/day PFOS (corresponding to >100 ppm serum PFOS) PFOS or PFOA orally for 6 months had decreased total serum cholesterol (Seacat et al., 2002). Some recent studies have begun to evaluate the effect of emerging, short-chain PFAS on metabolic disease and hepatic injury in mice (Wang J. et al., 2017; Chappell et al., 2020). In 2017, Wang et al. reported that daily administration of 1 mg/kg GenX for 3 weeks resulted in hepatomegaly, altered hepatic lipid metabolism gene levels, and hepatic injury (Wang J. et al., 2017). In early 2020, mice exposed to GenX at 0.5 and 5 mg/kg daily for 90 days demonstrated hepatic hypertrophy and hepatic lesions, upregulated fatty acid metabolism genes, and induced hepatic PPARα signaling (Chappell et al., 2020). These results are consistent with one of the well-established toxicodynamic mechanisms of PFAS, by which they can activate signaling pathways via PPARα (Intrasuksri et al., 1998; Takacs and Abbott, 2007; Rosen et al., 2008). Takacs et al. demonstrated the ability of PFAS, using transient transfection cell assays, to differentially bind and activate PPARα, β/δ, and γ (Takacs and Abbott, 2007). Activation of PPARα-signaling pathways leads to increased fatty acid oxidation and inhibition of the secretion of cholesterol and other lipids from the liver, resulting in a decrease of the overall levels of circulating lipids (Kennedy et al., 2004; Buhrke et al., 2015). The inconsistencies between the hyperlipidemia reported in human studies and the hypolipidemia observed in animal studies may be explained by interspecies differences or genetic influences. For example, Nakamura et. al. demonstrated that PFAS activate PPARα to a greater degree in mice than in humans (Nakamura et al., 2009). In these studies, wild-type mice (mPPARα), Pparα-null mice and humanized PPARα (hPPARα) mice were exposed to control, 0.1, or 0.3 mg/kg PFOA for two weeks. At the high dose, hepatic triglyceride levels, cholesterol levels, and PPARα-target genes were increased only in the mPPARα group, suggesting human PPARα to be less responsive to PFAS than mouse PPARα. In addition, expression of PPARα in humans is thought to be 1/10 that expressed by rodents (Palmer et al., 1998). Differences in lipoprotein metabolism also hinder extrapolation of data between rodents and humans, such as the absence of cholesteryl ester transfer protein (CETP) and a faster clearance of Apolipoprotein B (ApoB) from the liver, resulting in a higher proportion of HDL-C to LDL-C compared to humans (Princen et al., 2016). Because of these differences, some studies have been conducted using genetically engineered animal models that more closely mimic humans (Nakagawa et al., 2012; Pouwer et al., 2019).

**Table 2:** Key physiological endpoints from studies on PFAS exposure in rodent models related to metabolic disease.

**Citation**	**Species**	**PFAS**	**Exposure**	**Physiological endpoints**
Loveless et al. (2006)	mice (male Crl:CD®-1(ICR)BR); rats (male Crl:CD® (SD)IGS BR)	APFO	Oral gavage; 0, 0.3, 1, 3, 10, or 30 mg/kg; 14 days	↓ Total cholesterol ↑ Triglycerides (low doses)
Martin et al. (2007)	Rats (Spraque Dawley male)	PFOA and PFOS	Oral gavage; 20 mg/kg/day (PFOA) or 10 mg/kg/day (PFOS); 1, 3, or 5 consecutive days	↓ Total cholesterol ↓ Cholesterol biosynthesis genes ↑ Hepatocellular hypertrophy
Nakamura et al. (2009)	Mice (129/Sv wild-type, Pparα-null, and humanized PPARα)	APFO	Oral gavage 0.1 or 0.3 mg/kg/day; 2 weeks	↓ Hepatic triglyceride and cholesterol (mPPARα only) No effect plasma total triglyceride or cholesterol
Qazi et al. (2010)	mice (C57BL/6 male)	PFOA and PFOS	dietary exposure; 0.002% (w/w) PFOA or 0.005% (w/w) PFOS; 10 days	↓ Total cholesterol ↓ Triglycerides (low doses) ↑ Hepatocellular hypertrophy
Nakagawa et al. (2012)	Mice (129/Sv wild-type, Pparα-null, and humanized PPARα)	APFO	Oral gavage; 1.0 and 5.0 mg/kg/day APFO; 6 weeks	↑ Hepatic triglyceride (Pparα-null and hPPARα) ↓ Hepatic triglyceride (mPPARα) ↑ Steatosis
Butenhoff et al. (2012)	Rats (Spraque Dawley male and female)	PFOS	Dietary exposure; 0, 0.5, 2, 5, and 20 μg/g (ppm) diet for up to 104 weeks	↓ Total cholesterol ↑ hepatocellular hypertrophy
Han et al. (2018)	Rats (Spraque Dawley male)	PFOS	Oral gavage; 1 or 10 mg/kg/day; 28 days	↓ Hepatic oxidative stress ↑ hepatic apoptosis

### Non-alcoholic Fatty Liver Disease

Epidemiological studies in humans have linked PFAS exposure to fatty liver disease. The liver provides the principal line of defense against harmful xenobiotics, making it the organ most frequently affected by industrially- and commercially- manufactured chemicals. After entrance into the body through dietary exposure, PFAS proceeds to the liver. Due to structural similarities between fatty acids and PFAS, PFAS are able to bind to liver-fatty acid binding protein (L-FABP) for transport into hepatocytes and their nucleus (Luebker et al., 2002; Sheng et al., 2018). Once in the nucleus, PFAS are able to interfere with the DNA and transcriptional pathways. PFAS can further proceed to be a component of bile (Genuis et al., 2010). In the bile, it remains within the enterohepatic circulation pathway and eventually accumulates in the liver. Chronic exposure to PFAS may lead to injuries to the liver cells and increase risks for non-alcoholic fatty liver disease (NAFLD). NAFLD is characterized by excessive fat accumulation in the liver unrelated to significant alcohol consumption. NAFLD has been more specifically defined as macrovesicular or microvesicular accumulation of triglycerides in at least 5% of hepatocytes (Aly and Kleiner, 2011; Kawano and Cohen, 2013). NAFLD is the most predominant liver disease in humans, with a prevalence rate of 30% in the U.S. adult population (Estes et al., 2018). Fatty liver disease incorporates a spectrum of liver pathologies that can progress from simple fatty liver to steatohepatitis (NASH) which is characterized by increased fibrosis and inflammation. NASH can sometimes end in cirrhosis and hepatocellular carcinoma (Cohen et al., 2011; Kleiner and Brunt, 2012). Studies involving populations exposed to PFAS-contaminated water sources revealed strong associations between serum concentrations of PFOS and PFOA with elevated ALT levels, a surrogate marker for fatty liver disease (Gallo et al., 2012; Darrow et al., 2016). Analysis of the general U.S. adult population, based on NHANES data, also established an association between elevated ALT levels and elevated serum PFAS concentrations (Lin et al., 2010; Gleason et al., 2015; Jain and Ducatman, 2019b). However, there have been some studies debating the nuances of this association. A U.S. pregnancy cohort study reported that children exposed to higher levels of PFAS had lower ALT levels, revealing the need for further studies and analysis (Mora et al., 2018). Additional markers of liver injury, such as the liver apoptosis marker cytokeratin 18, were reported by Bassler et al. to be positively associated with PFAS levels as well (Bassler et al., 2019). Interestingly, this study also found PFAS exposure to be associated with decreased inflammation. In addition, a recent study by Jin et al. revealed that higher exposure to PFAS was associated with more severe fatty liver disease in children 7–19 years old based on liver biopsy histology scored for steatosis (Jin et al., 2020). In this study, PFAS exposure was associated with increased risk of steatohepatitis, fibrosis, lobular inflammation, and increased NAFLD score. Furthermore, this study found higher concentration of plasma PFAS to be associated with alterations in key metabolic pathways, including glycerophospholipid and tyrosine metabolism, that may be impacting fatty liver disease severity. Both dysregulated tyrosine metabolism and dysregulated glycerophospholipid metabolism have been previously associated with the severity of NAFLD progression (Gorden et al., 2015; Jin et al., 2016). In addition, cell and animal-based studies have reported an increased incidence of oxidative stress characterized by increase production of reactive oxygen species (ROS) after exposure to PFAS through dietary pathways (Wielsøe et al., 2015; Wang X. et al., 2017; Han et al., 2018), which can lead to mitochondrial dysfunction and consequently induce cell apoptosis. Excessive ROS production and apoptosis can increase the susceptibility to NAFLD. Although the specific mechanisms leading to the association of PFAS levels with NAFLD remain unknown, other human cross-sectional studies have reported that PFAS exposure was associated with dysregulation of fatty acid metabolism in children, adult, and elderly populations, even at low levels of PFAS exposure (Wang J. et al., 2017; Alderete et al., 2019; Kingsley et al., 2019; Salihovic et al., 2019).

It should be noted that genetic variants play an important role in the development of NAFLD/NASH in humans. A few single nucleotide polymorphisms and their associated genes have been identified in genome-wide association studies of NAFLD, e.g., PNPLA3, TM6SF2, HSD17B13, MBOA7, PPP1R3B, LYPLAL1, and GCKR (Liu Z. et al., 2020). Therefore, the inter-individual differences as well as the inter-study discrepancies in the impact of PFAS on NAFLD risk may be attributed to these genetic variations. More studies are needed to further clarify this PFAS-genetic interaction in the development of NAFLD.

## Dietary Modulation of PFAS Toxicity

Studies over the past several decades in animals and humans have demonstrated the range of health effects linked to PFAS exposure. Because of this, it is important to gain a better understanding of the mechanisms involved in PFAS-induced manipulation of lipid metabolism. Studies using rodents are key to learning these mechanisms and how they can be translated to humans. However, in most published rodent studies, the animals have been fed a standard diet, containing low fat and negligible cholesterol. Only a few studies have examined the effects of PFAS exposure in combination with a diet similar to one consumed by a majority of humans in western societies that is high in fat and cholesterol. Table 3 outlines some of these important studies on PFAS exposure in animal models and the resulting endpoints related to the interactions between diet and metabolic disease. Previous studies in humans have suggested that plasma cholesterol and PFAS serum concentrations are directly related, while rodent studies suggest an inverse relationship when fed a standard diet. To examine the effects of dietary modulation of PFAS toxicity in a mouse model, Rebholz et al. fed mice a PFOA-laced Western diet (0.25% cholesterol, 32% fat) for 6 weeks (Rebholz et al., 2016). PFOA exposure led to hypercholesteremia in both male and female mice compared to control, in contrast to previous studies using a standard diet. Plasma cholesterol levels in mice exposed to PFOA increased by 70% in females and a smaller 35% increase in males. Liver weights was increased in mice fed PFOA, but hepatic tissue cholesterol levels were largely unchanged. Of note, the increase in plasma cholesterol was determined to occur in the HDL fraction, while previous studies in humans have shown that PFOA-induced hypercholesteremia is usually the result of higher LDL and lower HDL cholesterol levels (Olsen and Zobel, 2007; Steenland et al., 2009; Frisbee et al., 2010; Geiger et al., 2014). However, several high fat diet studies have reported that exposure to PFAS in mice fed a high fat diet generally resulted in increased hepatic steatosis and hypolipidemia. Male BALB/c or C57BL/6 mice exposed to PFOS or PFOA on a standard or high fat diet had reduced serum cholesterol and triglycerides, as well as increased hepatic injury and steatosis (Tan et al., 2013; Wang et al., 2014). These changes were found to occur mainly through inhibited excretion of low-density lipoproteins. A recent study in 2019 by Pouwer et al. explored the effects of PFOA on plasma cholesterol and triglyceride metabolism using genetically engineered APOE^*^3-Leiden.CETP mice, which exhibit human-like lipoprotein metabolism through cholesteryl ester transfer protein expression and delayed apoB clearance (Pouwer et al., 2019). In this study, mice fed a Western type diet for 4 weeks were then exposed to varying concentrations of PFOA incorporated into the Western diet (0.25% cholesterol, 14% fat) for 4 or 6 weeks. Mice exposed to PFOA had decreased plasma triglycerides, decreased plasma total cholesterol, and decreased non-HDL cholesterol, whereas HDL cholesterol was increased (Pouwer et al., 2019). These changes were mediated mainly through activation of PPARα. Although no difference from control was seen in hepatic free cholesterol or triglycerides, PFOA exposure did induce hepatic hypertrophy and microvesicular steatosis. Similar decreases in plasma lipoproteins were reported in mouse experiments using APOE^*^3-Leiden.CETP mice were fed a Western-type diet and exposure to PFBS, PFHxS, or PFOS for 4–6 weeks (Bijland et al., 2011). In this study, exposure to PFAS resulted in reduced plasma triglycerides, total cholesterol, non-HDL-C and HDL-C. These reductions were largely the result of impaired lipoprotein production via activation of PPARα and pregnane X receptor. PFAS exposure also led to increased hepatic steatosis, displaying increased liver weight and hepatic triglyceride content. A recent study by Marques et al. demonstrated that PFOS exacerbates hepatic steatosis in mice fed a high-fat diet (Marques et al., 2020). Further mRNA and proteomic analysis revealed PFOS-induced lipid and xenobiotic transporters and metabolism pathways that interfered with lipid loss when mice were switched from a high-fat diet to a standard diet and exacerbated the lipid accumulation in mice on a high-fat diet. In addition, other studies have shown that exposure to PFOS in conjunction with a high fat diet increases atrophy and accumulation of adipocytes in immune organs such as the thymus and spleen (Wang et al., 2011). Thus, PFAS can lead to dysfunction of immune organs via interference with lipid metabolism, leading to senescence and the attenuation of humoral immune responses that play a role in systemic metabolic homeostasis (Zmora et al., 2017).

**Table 3:** Key physiological endpoints from studies on PFAS exposure in rodent models related to dietary-modulation of metabolic disease.

**Citation**	**Species**	**PFAS**	**Exposure**	**Physiological endpoints**
Bijland et al. (2011)	Mice (APOE*3-Leiden.CETP male)	PFBS, PFHxS, or PFOS	Western-type diet; dietary exposure PFBS, PFHxS, or PFOS (30, 6, and 3 mg/kg/day, respectively); 4–6 weeks	↓ Plasma triglycerides and total cholesterol ↑ Steatosis
Tan et al. (2013)	Mice (C57BL/6N male)	PFOA	Low- or high-fat diet; dietary exposure 5 mg/kg/day PFOA; 3 weeks	↑ Steatosis ↑ liver injury
Wang et al. (2013)	mice (BALB/c male)	PFOA	Regular or high-fat diet; oral gavage 0, 5, 10, or 20 mg/kg/day PFOA; 14 days	↓ Plasma triglycerides and total cholesterol ↑ Steatosis
Wang et al. (2014)	Mice (BALB/c male)	PFOS	Regular or high fat diet; oral gavage 0, 5, or 20 mg/kg PFOS; 14 days	↑ Plasma cholesterol ↑ Steatosis
Rebholz et al. (2016)	Mice (C57BL/6 and BALB/c)	PFOA	Western-type diet; dietary exposure 0.56 mg/kg/day; 6 weeks	↑ Plasma cholesterol
Huck et al. (2018)	Mice (C57BL/6J male)	PFOS	Regular or high-fat diet; dietary exposure 1 mg/kg PFOS; 6 weeks	↓ Steatosis
Li et al. (2019)	Mice (C57BL/6 male)	PFOA	Regular or high-fat diet 16 weeks; oral gavage 1 mg/kg/day PFOA for additional 2, 8, or 16 weeks	↓ Steatosis
Pouwer et al. (2019)	Mice (APOE*3-Leiden.CETP male)	PFOA	Western-type diet; dietary exposure 10, 300, 30,000 ng/g/day; 4–6 weeks	↓ Plasma triglycerides and total cholesterol (30,000 ng/g/d PFOA) ↑ Steatosis (10 and 300 ng/g/d PFOA) No effect plasma lipid levels (10 and 300 ng/g/d PFOA)

Nevertheless, the ability of PFAS exposure simultaneous with a high-fat diet to cause hypolipidemia and increase hepatic steatosis remains a disputed topic. A study conducted by Wang et al. looked at male BALB/c mice exposed to 5–20 mg/kg PFOA via oral gavage for 14 days while being fed either a regular or high-fat diet (Wang et al., 2013). In this study, it was reported that the decreased levels of plasma lipoproteins and changes in hepatic histology were not significantly different in high fat diet vs. regular diet. Furthermore, other recent studies have shown that PFAS actually have a protective effect against high-fat diet-induced hepatic steatosis. Huck et al. reported that although a high-fat diet alone or 1 mg/kg daily PFOS treatment alone for 6 weeks led to increased hepatic steatosis, this increase was prevented through a combined high-fat diet and PFOS treatment (Huck et al., 2018). Furthermore, when mice were fed a high-fat diet for 16 weeks to induce steatosis, subsequent administration of 1 mg/kg PFOA daily by oral gavage for an additional 2–16 weeks was shown to decrease the severity of hepatic steatosis (Li et al., 2019). Transcriptomic analysis revealed that the preexisting steatosis led to enhanced PFOA-related lipid oxidation pathways in the mice fed a high fat diet. Although further studies are needed, this model takes into consideration the effects of PFAS on preexisting and naturally progressing NAFLD and may be more relevant to actual human exposure conditions, specifically in overweight individuals. To date, very few human studies of PFAS toxicity have examined the role of diet as a modulatory factor. In 2018, Liu et al. analyzed the ability of PFAS to interfere with body weight control in humans via data from the 2-year POUNDS Lost randomized clinical trial (Liu G. et al., 2018). In the POUNDS Lost trial, 621 overweight and obese participants went through a 6-month weight loss period, followed by a 6–24 month weight regain period. Higher plasma PFAS concentrations were found to be associated with greater weight regain, especially in women. The higher plasma PFAS levels and greater weight regain were also associated with a slower regression of resting metabolic rate during the weight regain period. These data illustrate an interaction between PFAS and dietary regulation in humans. Furthermore, in a 2019 study analyzing data from NHANES for 2005–2014, it was demonstrated that obesity in both males and females altered the cross-sectional associations of different PFAS with plasma lipid concentrations (Jain and Ducatman, 2019a). In this study, both PFOA and PFNA were positively associated with total cholesterol levels for obese males, but not for non-obese males. Additionally, PFDA and PFNA were positively associated with total cholesterol levels for obese females, but not for non-obese females. Overall, these findings suggest a greater vulnerability in obese humans to higher cholesterol levels, with some variances between men and women. However, the impact of dietary modulation on PFAS toxicity in humans remains a largely unexplored area of research and warrants further investigation to better understand the interactive effects of diet and PFAS toxicity.

## Future Directions and Conclusions

**Figure 2:**
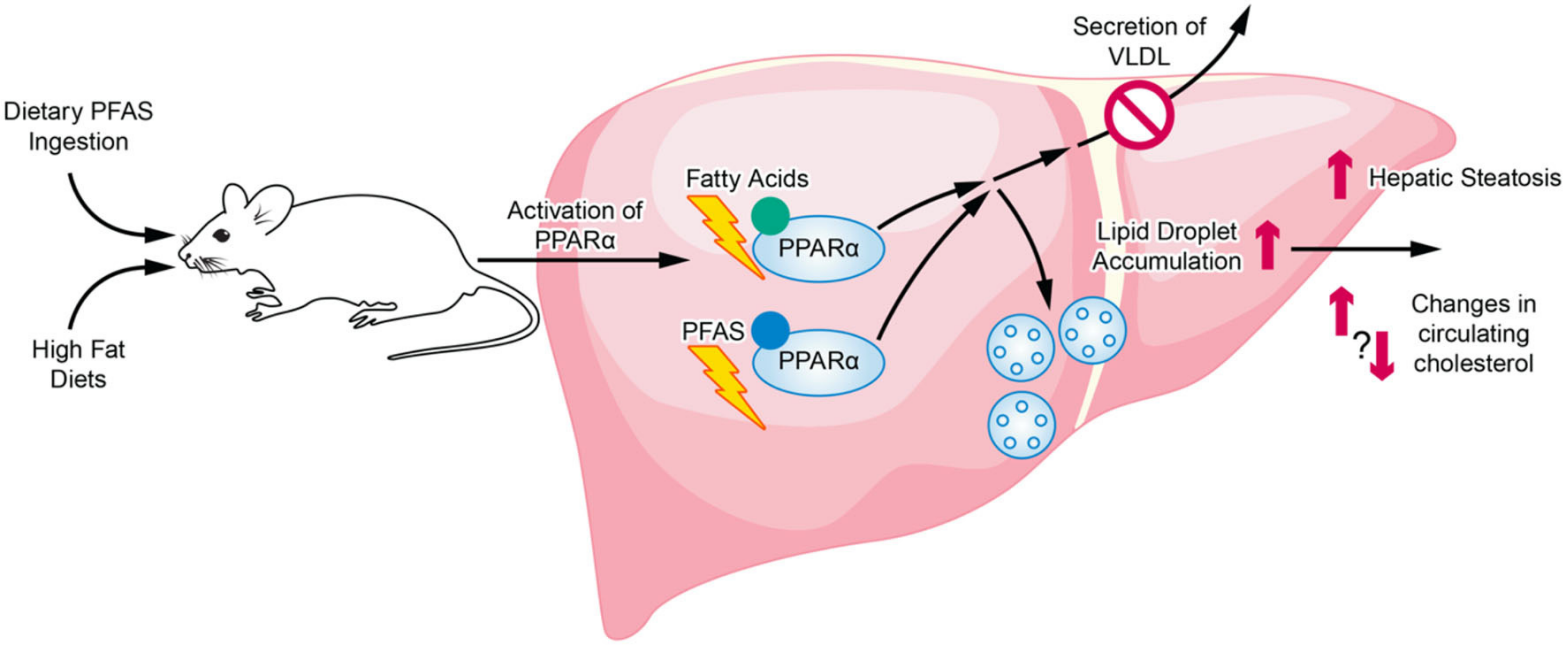
Overview of PFAS and HFD interaction in relation to activation of PPARα and regulation of hepatic lipid accumulation and secretion. The structurally similar PFAS chemicals and dietary fatty acids can both activation PPARα. Sustained PPARα activation eventually can lead to the inhibition of lipid secretion into the blood as well as an increase in lipid droplet accumulation in the liver.

Although current animal studies highlight a relationship between diet and PFAS exposure, inconsistencies exist among the studies on the changes in lipid metabolism and cardiometabolic profiles that are produced. Numerous studies have highlighted the association between PFAS exposure in humans to hyperlipidemia and fatty liver disease, while studies in rodents conversely report hypolipidemia. When diet is further introduced as a variable, most rodent studies investigating mice exposed to PFAS and a high fat diet reported an exacerbation of results found in standard diet studies, namely increased hepatic steatosis and hypolipidemia. These studies varied across the mouse strains and models used (Tan et al., 2013; Wang et al., 2014; Pouwer et al., 2019). However, a few studies have shown that PFAS actually have a protective effect against high fat diet-induced hepatic steatosis (Huck et al., 2018; Li et al., 2019). Although the exact cause of these inconsistencies is not known, important differences between these studies include whether steatosis was preexisting before PFAS exposure as well as the length of the study. Furthermore, there are many limitations in the effort to extrapolate health effects observed in laboratory animals to humans. Many animal studies report that PFAS, which are structurally similar to fatty acids, act in major part through PPARα, a key transcription factor regulating lipid metabolism (Rosen et al., 2008; Wolf et al., 2008; Bjork et al., 2011). Several studies reporting on the exacerbation of PFAS exposure effects by high fat diet also attribute the effects primarily to mechanisms involving PPARα activation (Tan et al., 2013; Li et al., 2019; Pouwer et al., 2019). The role of both dietary PFAS and the consumption of a high fat diet on PPARα activation and the downstream effects on hepatic lipid accumulation and secretion are illustrated in Figure 2. However, important species differences exist between PPARα expression and its induced effects in rodents compared to humans that are problematic for the extrapolation of results from animal studies to humans (Palmer et al., 1998; Cattley, 2004; Lake, 2009). Moreover, human PPARα transgenic mice have been generated and studies report differences in fenofibrate activation of hepatic lipid metabolism and proliferation responses between human PPARα transgenic mice and wild-type mice (Yang et al., 2008). Further investigations utilizing human PPARα transgenic mice are needed to better exploit the utility of animal studies in exploring the role of PPARα in human-relevant metabolic syndrome and NAFLD models. However, although PPARα activation is often identified as a major pathway, PFAS exposure has also been shown to produce PPARα-independent effects through receptors such as the pregnane X receptor and the farnesoid X receptor (Rosen et al., 2010; Bjork et al., 2011). Further studies are required to examine the multiplicity and relative effects these different pathways induced by PFAS exposure. In addition, in a comprehensive review of the literature surrounding PFAS toxicokinetics in both animals and humans, interspecies differences have been identified, including species-specific tissue distribution, half-life, and maternal transfer. For example, humans may be less susceptible to hepatic effects from PFAS exposure than rodents since most PFAS (aside from PFOA) preferentially distribute to the kidneys in humans as opposed to preferential distribution to the liver in rodents (Pizzurro et al., 2019). Furthermore, there are also important differences between specific PFAS, such as varying chain lengths and toxicological modes of action, that must be studied at greater depth in order to more confidently establish human-relevant findings from animal study data (Pizzurro et al., 2019). Since the 2000's, long-chain PFAS are being replaced by shorter-chain, emerging PFAS, such as GenX (Brendel et al., 2018). The main rationale for the transition from long-chain, legacy PFAS to the shorter-chain PFAS was that shorter-chain PFAS are considered less toxic and less bioaccumulative. However, recent findings have brought into question the actual toxicity of these emerging PFAS as well as environmental and regulatory concerns (Gebbink et al., 2017; Brendel et al., 2018; Gomis et al., 2018). Overall, few studies exist for these new, emerging PFAS, or PFAS mixtures that include both legacy and emerging contaminants of concern, especially those examining human-relevant health ramifications. The toxicity of these emerging PFAS, and the possible dietary modulation of that toxicity, remain largely unexplored and calls for additional studies to provide greater insight.

## Summary and Concluding Remarks

The PFAS family of synthetic chemicals are pervasive throughout the environment and exposure is known to occur via multiple routes, including indoor dust and air, drinking water, and diet. PFAS exposure is associated with hyperlipidemia in humans, while animal studies commonly report hypolipidemia. Furthermore, epidemiological studies in humans have linked PFAS exposure to fatty liver disease. Several recent studies exploring dietary modulation of PFAS toxicity report an exacerbation of hepatic steatosis and hypolipidemia. However, the specific interactions of diet with PFAS toxicity remains unclear, and some studies have shown PFASs to have a protective effect against high-fat diet-induced hepatic steatosis, especially when the condition is pre-existing. The manufacture of long-chain PFAS has been halted because of substantial public and media attention due to their documented water pollution and toxicity in the U.S., European Union, and Australia. However, regulations on PFAS substances in other areas of the world are absent, and information regarding PFAS contamination and exposure levels in many nations is lacking. Furthermore, PFAS contamination can negatively impacts humans in more ways than simply direct human exposure. The ubiquitous nature of PFAS in the environment has also been shown to disturb natural ecosystems by disrupting aspects of animal development, life cycles, and reproduction. While companies have halted the manufacture of long-chain PFAS, short-chain emerging PFAS, such as GenX, are being produced in their place. Toxicities of short-chain PFAS are not well-understood and more studies are required in order to evaluate the risks associated with them. Despite increased awareness and documentation, both long- and short-chain PFAS remain present in drinking water sources worldwide, and regulators in nations across the globe may need to reexamine risk assessment and toxicity studies in order to accurately evaluate the NOAEL and LOAEL for human populations.

## Author Contributions

KR and ZI wrote and edited the manuscript. MP and WL wrote, edited, and oversaw the completion of the review. All authors have read and approved of the manuscript.

## Conflict of Interest

The authors declare that the research was conducted in the absence of any commercial or financial relationships that could be construed as a potential conflict of interest.
